# Imaging-genetics-based dementia risk prediction using deep survival neural networks in the Rotterdam Study

**DOI:** 10.21203/rs.3.rs-7622942/v1

**Published:** 2025-09-23

**Authors:** Jing Yu, Mathijs T. Rosbergen, Frank J. Wolters, Esther E. Bron, Meike W. Vernooij, M. Arfan Ikram, Gennady V. Roshchupkin

**Affiliations:** Erasmus MC University Medical Center; Erasmus MC University Medical Center; Erasmus MC University Medical Center; Erasmus MC University Medical Center; Erasmus MC University Medical Center; Erasmus MC University Medical Center; Erasmus MC University Medical Center

**Keywords:** dementia risk prediction, brain MRI, genetics, deep learning, survival analysis, cohort study

## Abstract

Accurate dementia risk prediction is challenging, and may be facilitated by better use of imaging and genetic data, including their complex interactions. We explored using deep survival neural networks to integrate these multi-modal, high-dimensional data. We included 3521 Rotterdam Study participants, 6340 magnetic resonance imaging (MRI) scans, with follow-up clinical diagnosis for dementia, and used 504 samples from Alzheimer’s Disease Neuroimaging Initiative (ADNI) as an external validation. Genetic data included APOE-ε4 status and 76 additional SNPs. We developed models combining Convolutional Neural Networks (CNN) and Cox Proportional Hazards (CPH) models and provided post-hoc explanations. Our models outperformed CPH models including age, sex, and genetic inputs in both Rotterdam Study and ADNI by C-index of 0.88/0.63 V.S. 0.85/0.58, p-value of 0.02/0.002. Although their performance did not surpass CPH models also included MRI markers (0.89/0.66), additional predictability was obtained in age-stratified prediction in ADNI. Incorporating CNN image features in CPH models further increased performance to highest C-index of 0.90/0.69. Age and image had the highest importance in prediction, with age, image and genetic features showing the strongest interactions. Our approach indicates that imaging and genetic data can be feasibly integrated for dementia risk prediction, with informative extraction, reliable explanations and potential predictive gains.

## 1. INTRODUCTION

Most forms of dementia are characterized by incurable and irreversible cognitive impairment [1]. Consequently, accurate early risk prediction takes on significant importance, as it can enable timely interventions that ultimately enhance the quality of life for both individuals at risk and their caregivers. However, predicting dementia remains challenging due to the complexity of the disorder. Early studies that focused on demographic and basic health measures showed limited predictive power beyond age [2], while lately the advances of imaging and high-throughput omics technologies have revolutionized this field, enabling large-scale population studies with extensive imaging and genetic datasets.

Brain imaging and genetic data have each contributed valuable insights into dementia. For instance, brain magnetic resonance imaging (MRI) measurements such as gray matter (GM) volume [3, 4], shape of subcortical structures [5], and cortical thickness [6] have been identified as important biomarkers for dementia. Similarly, genetic variations including the *APOE*-ε4 allele [7] and various single nucleotide polymorphisms (SNPs) [8–10] have been found associated with Alzheimer’s Disease. Concurrently, numerous prediction models have been developed, many of which leveraging survival models like cause-specific Cox Proportional Hazards (CPH) models and sub-distribution hazard models [11–12]. By incorporating such imaging and genetic risk factors, these models have shown improved predictive power [13]. While survival analysis is well-suited for risk prediction tasks, particularly its ability to handle time-to-event data and right-censored samples [14], these conventional methods rely on linear assumptions that often invalid in real-world scenarios. To address this limitation, neural network survival models have been subsequently proposed, representing nonlinear extensions of CPH models [15–16]. Recent dementia risk prediction studies have applied these models to dozens or hundreds of features, such as GM volumes from regions of interest (ROIs) [17], as well as imaging and genetic features extracted from classification models [18, 19], which have shown better performance than CPH models with the same inputs.

Current models have extended the predictive ability, yet have predominantly relied on pre-defined regions or pre-extracted features within each individual modality [17–19]. This reliance poses several drawbacks: selecting and extracting features can be time-consuming and computationally expensive; the performance heavily depends on the quality of extracted features; and features like volumes are limited in representing highly abstract information [20]. In the same time, extracting features from isolated modalities overlooks the complex interweaving between imaging and genetic data, which may aid in predicting their combined influence on the disease progression. Advances in the field of imaging genetics revealed that genetics strongly impacts brain structures [21–23], with patterns varying across image measurement levels (e.g., volume, thickness and surface area [24]) and extending beyond anatomical defined regions (e.g., to voxel-level [21]). This highlights the immense potential of integrating full images and genetic data for predicting dementia. However, at the same time, imaging and genetic data typically exhibit high-dimensionality and distinct data structures, posing significant challenges for a single end-to-end model. With the development of deep learning (DL) models, more advanced DL techniques including dropout regularization, bayesian optimization etc., have been applied to build more sophisticated deep survival models [16], e.g., to include large-scale genomic data [25]. Prior work in cancer risk prediction has shown the feasibility of deep survival models accommodating 2D histological images using convolutional neural networks (CNNs) when incorporating two genomic variables [26]. While promising, there has been no endeavor in dementia risk prediction that fully integrates these advanced techniques.

In this work, we trained 3D CNN-based deep survival neural networks to leverage high-dimensional 3D brain MRI images alongside genetic data integration on the large population-based Rotterdam Study, and used the Alzheimer’s Disease Neuroimaging Initiative (ADNI) as an external validation to assess generalizability. Furthermore, understanding how models work is crucial for safe practice, especially in the medical domain. Therefore, we also provide post-hoc explanations of our models. We focus on two primary research questions: a) Can deep survival models improve prediction of dementia in comparison to conventional CPH models? b) What are the patterns within and between imaging, genetic and other data modalities that drive the risk prediction in deep survival model?

## 2. RESULTS

### 2.1. Population characteristics

Participants’ dementia diagnoses, time-to-event, demographics, genetic data and brain MRI markers used in the model computation and comparison are in Table 1. We report descriptive statistics for the total samples and for the training, validation, and test sets from the first fold of the 5-fold cross-validation in Rotterdam Study. In addition, statistics for the external validation set from ADNI are provided. The statistics of variables are calculated based on the number of images (N_img_). All the volumetry MRI markers are adjusted for intracranial volume, WM hyperintensity volume is further log-transformed due to the skewed distribution.

Among the Rotterdam Study samples, over the course of average 7.75-year follow-up, 7.33% (258/3521) of the participants (6.09% (386/6340) of the images) developed dementia, with an average scanning age of 68.74 years and 28% carrying *APOE*- ε4 variation. Similar variable distributions were maintained in the training, validation and test sets. As ADNI carefully selected participants based on their disease status, ADNI samples are generally with elder age, higher genetic risk and higher dementia rates. 33% (170/515) of the participants developed dementia in average 8.17-year follow-up, with an average scanning age of 75.36 years and 46% carrying *APOE*- ε4 variation. More details of data characteristics including the variable distribution plots and dementia conversion curves are displayed in **S1–2**.

### 2.2 Model performance

#### 2.2.1 Model comparisons

Model performance measures of dementia risk prediction on Rotterdam Study test set are presented in Table 2A. Generally, the baseline CPH models using only demographic and genetic data could reach quite good performance (C-index > 0.8). Adding T1 volumetry MRI markers (i.e., total brain volume and hippocampal volume) to the linear CPH models improved the prediction C-index significantly by 0.03–0.04, while including all MRI markers (additional WM hyperintensity volume and presence of lacunes/cortical infarcts) only further increased C-index marginally by 0–0.01. Contrary to expectations, the DeepSurv models, representing the nonlinear version of the extended linear CPH models with all MRI markers, didn’t show better performance than CPH Models. As for our deep survival models, using MRI images could only achieve the intermediate performance between baseline CPH models and the extended CPH models with MRI markers in Model 2–4. Our models achieved significant better performance than baseline CPH models, with C-index difference ranging from 0.02 to 0.05, p-values ranging from 0.003 to to 0.05. Our models achieved slightly improvement than the CPH models in model 5, which includes high-dimensional genetic data (SNPs). The models trained with the proposed fine-tuning strategy achieved slightly, though not significantly, better performance compared to those trained from scratch.

Although our deep survival models with MRI images did not improve the prediction than linear CPH models with MRI markers, the MRI images appeared to take the complementary role to MRI markers in providing predictive information. Combining image features from our deep survival (fine-tuned) models with MRI markers showed the best performance in the all Model 1–5, and the improvement to extended CPH models is significant in external validation (see Table 2B), with C-index difference ranging from 0.01 to 0.04, p-values ranging from 0.004 to to 0.01.

As for demographic and genetic inputs in addition to MRI inputs, sex and age provided significant addition predictive value to MRI markers but not MRI images. Genetic inputs contributed to the prediction additionally in all scenarios, with *APOE*-ε4 (Model 3) improving the C-index by 0–0.02, and the addition PRS (Model 4) further increasing it by 0–0.01. Model 4 with *APOE*-ε4 and PRS presented the best performance overall, with highest C-index of 0.90 when combining image features and MRI markers, C-index of 0.88 in deep survival models, 0.85 and 0.89 in baseline and extended CPH models. The input of raw SNPs (Model 5) however, did not offer improvement over *APOE*-ε4 (Model 3). While it deteriorated the performance of linear CPH models, comparable performance was still observed in deep survival models.

External validation on ADNI showed significantly lower C-index (C-index < 0.7), which is reasonable considering the heterogeneity between the two study samples. Nevertheless, most of the performance patterns observed from the Rotterdam Study test set generalized well to the ADNI cohort.

#### 2.2.2. Reproducibility

We computed the prediction outcomes with paired scan-rescan measures taken in a short period and obtained an intraclass correlation coefficient (ICC) of 0.973. In **Figure S4**, we show that most of the dots representing paired scans were closely aligned with the diagonal. Hence, these results suggest that our model was highly reproducible.

#### 2.2.3 Stratified analyses

**Figure 1:**
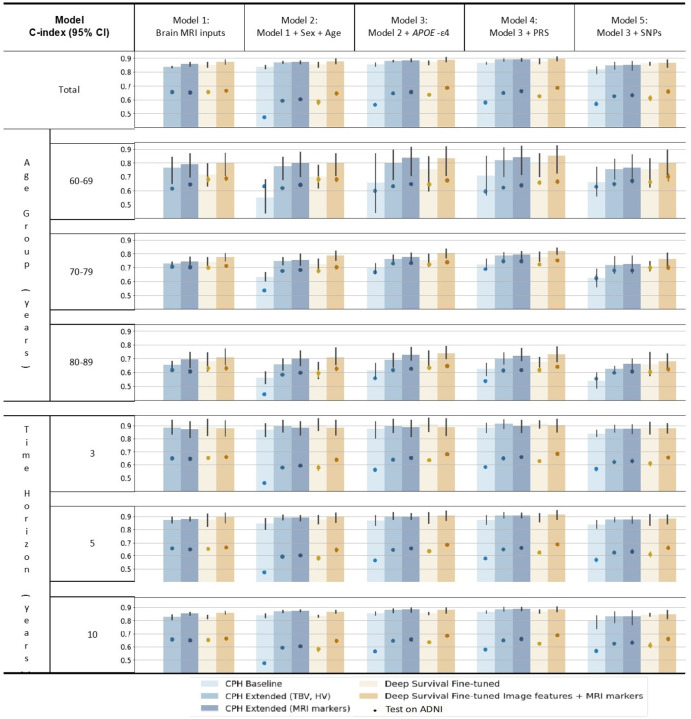
Stratified prediction performances on different age groups and follow-up time horizons of linear CPH models and deep survival models. Mean C-index and 95% confidence intervals are based on 5-fold cross-validation. Acronyms: magnetic resonance imaging (MRI), polygenic risk score (PRS), single nucleotide polymorphism (SNP), Cox Proportional Hazards (CPH)

We computed the model performance across different age groups and follow-up time horizons, on models of different brain MRI, demographic and genetic inputs, as illustrated in Fig. 1. Generally, higher prediction C-index values were achieved in younger age groups. It is noticeable that after restricting to certain age groups, the performance of baseline CPH models with sex and age input (Model 2) dropped dramatically due to the controlled effect of age. However, the inclusion of MRI (extended CPH models and deep survival models) and genetic data (Model 3–5) helped to mitigate this drop, especially in younger subjects. Similarly, slightly higher C-index values were found over shorter follow-up times, while all models were quite robust across different time horizons.

Although the model performance difference on Rotterdam Study test set was not notable, significant difference could be found in external validation test on ADNI. Particularly, combining MRI markers with deep survival model image features together yielded significant best performance on ADNI dataset, in all the age groups and follow-up time horizons. Differences were observed between the test set results and those from the external validation set. For example, among 60–69 age group, our deep survival fine-tuned model exhibited deteriorate performance than extended CPH models on Rotterdam Study, but exhibited the best performance on ADNI.

### 2.3 Feature importance analysis

Although Model 5 (MRI images + Age + Sex + *APOE*-ε4 + SNPs) did not yield the best predictive performance, we performed explanation on Model 5 to evaluate the patterns of Image- and SNP-derived features identified by the deep survival models for dementia risk prediction and whether they are biologically consistent.

#### 2.3.1 Visualize voxel-level importance on image using Grad-CAM

**Figure 2:**
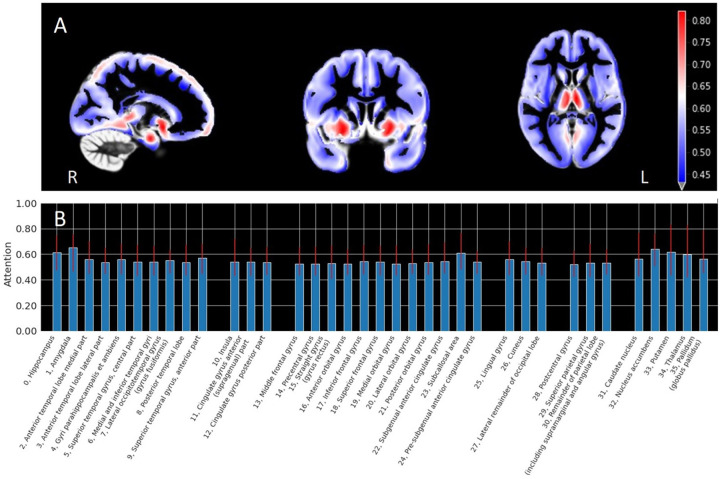
Attention intensity of the deep survival (trained from scratch) Model 5 (MRI images + Age + Sex + *APOE*-ε4 + SNPs). A. Heatmap on image slices on 3 different axes. B. Average intensity in brain regions. Temporal lobe: 0–9, Insula and cingulate gyri: 10–12, Frontal lobe: 13–24, Occipital lobe: 25 −27, Parietal lobe: 28–30, Central structures: 31–35. Acronyms: magnetic resonance imaging (MRI), single nucleotide polymorphisms (SNP)

Figure 2A visualizes the attention heatmaps of the CNN in the deep survival Model 5 on the average of all Rotterdam Study test samples, calculated using Grad-CAM. It shows three image slices projected onto the standard GM template on different axes. The left-right asymmetry could be seen in the attention map. Heatmaps on more image slices are shown in **Figure S8.1**.

Figure 2B displays the brain region importance calculated from the mean voxel attention intensity within each anatomically defined region, with the top 5 important regions: amygdala, nucleus accumbens, putamen, hippocampus and subcallosal area. More details of the brain region importance are shown in **Table S8.1**. The region importance calculated from one iteration (of the 5-fold cross-validation) were generally aligned with the average importance calculated from five iterations (see **Figure S8.2**), suggesting the robustness of importance explanation.

#### 2.3.2 Calculate feature importance using DeepSHAP

**Figure 3:**
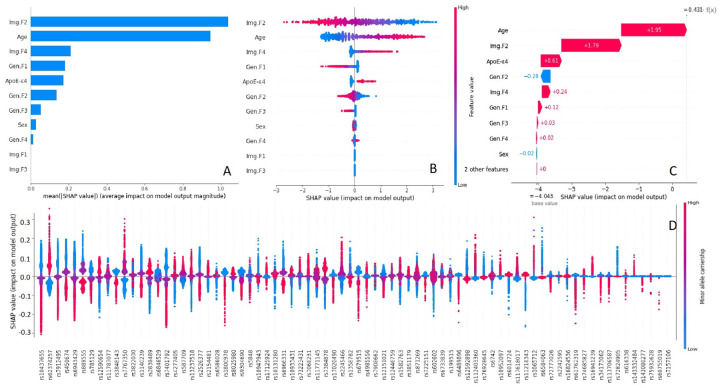
Model 5 (MRI images + Age + Sex + *APOE*-ε4 + SNPs). A. Ranked importance (mean absolute SHAP values) of all concatenated features. B. Feature attributions (SHAP values) of individual feature values, ranked by importance. Each dot represents a feature of an individual. Red indicates high feature value, and blue indicates low feature value. C. Individualized feature additive attributions (SHAP values), ranked by importance. Red indicates positive additive attribution, and blue indicates negative additive attribution D. Feature attributions (SHAP values) of individual SNP carrierships, ranked by importance. Red indicates high carriership of minor allele, and blue indicates low carriership of minor allele. Acronyms: Image Feature (Img.F), Genetic Feature (Gen.F), magnetic resonance imaging (MRI), single nucleotide polymorphism (SNP)

Figure 3A–B present the SHAP values of all concatenated features both on average (**3A**) and on individuals (**3B**), calculated from DeepSHAP. The features are ranked by feature importance (mean absolute SHAP values of all individuals). As shown in Fig. 3A–B, the network automatically assigned two important image features (Img.F2 and Img.F4) from GM MRI images for predicting dementia. In general, Img.F2 and age contributed the most to the risk prediction, followed with Img.F4, genetic feature 1 (Gen.F1), *APOE*-ε4, Gen.F2, Gen.F3, Sex and Gen.F4. We further plot the attention heatmaps of Img.F2 and Img.F4 based on Grad-CAM results (**Figure S8.3–4**), of which Img.F2 captured the main information compad to Fig. 2A, whereas Img.F4 also captured some additional information. Figure 3C provides an individualized explanation of the SHAP values, illustrating how each feature contributes additively to the model’s outcome. Figure 3D displays the SHAP values of SNPs on individuals ranked by importance. The importance calculated from one iteration (of the 5-fold cross-validation) were generally aligned with the average importance calculated from five iterations (see **Figure S9.1**), suggesting the robustness of importance explanation.

### 2.4 Feature interaction analysis

#### 2.4.1 Feature interaction strength

**Figure 4:**
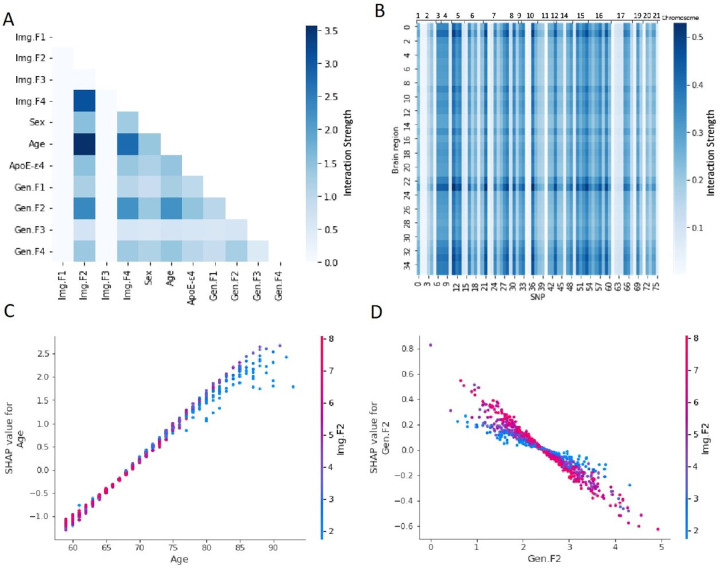
Feature interaction on deep survival (trained from scratch) Model 5 (MRI images + Age + Sex + *ApoE*-ε4 + SNPs). A. Interactionstrength between each pair of features. B. Mapped interaction strength of all the pairs of brain regions and single nucleotide polymorphisms (SNPs). The brain regions are ranked by structures. The SNPs are ranked based on chromosome and position. C. Interaction plots between Img.F2 and age. D. Interaction plots between Img.F2 and Gen.F2. Acronyms: Image Feature (Img.F), Genetic Feature (Gen.F), magnetic resonance imaging (MRI), single nucleotide polymorphism (SNP)

As depicted in Fig. 4A, the strongest interactions identified by statistical interaction detection method were between Img.F2, Img.F4 - age, Img.F2 - Img.F4, and Img.F2, Img.F4 - Gen.F2. The further mapped interaction strengths between image regions and SNPs are shown in Fig. 4B, with image regions ranked by structures (the same as in Fig. 2B) and the SNPs ranked by chromosome and position. The highest interaction for risk prediction between brain regions and SNPs were presented between subcallosal area (No.23) and rs112403360 (No.11). The interaction also exhibited similar patterns in adjacent brain regions and SNPs. For example, regions 31–35 in the central structures all showed comparable higher interaction strengths. Similarly, SNPs 61–64 on chromosome 17 exhibited similar lower interaction strengths.

#### 2.4.2 Feature interaction plot

The interaction plots with SHAP values between Img.F2 and age, Img.F2 and Gen.F2 (with relatively high interaction strengths displayed in Fig. 4A) are shown in Fig. 4C–D. More interaction plots between different features can be found in **Figure S9.6–9**. In Fig. 4C, the SHAP value for age showed a steeper slope when Img.F2 was higher, indicating that higher Img.F2 amplifying the contribution of age to the model prediction. Likewise, in Fig. 4D, interaction effect was present as the SHAP value for Gen.F2 showed a steeper slope when Img.F2 was higher. However, this effect was not clearly seen in the plot between Img.F2 and Gen.F4 (See **Figure S9.8**), which was inline with the lower calculated interaction strengths (Fig. 4A). Similar interaction patterns as Gen.F2 was also found on *APOE*-ε4 (**Figure S9.9**).

## 3. DISCUSSION

With the availability of extensive imaging and genetic data in large population study, building a more comprehensive risk prediction model is of great significance at this stage for facilitating dementia risk prediction. Prior work has mostly used derived features from brain images [13, 17–19] due to the challenge of high-dimensionality, and used a single data domain [17, 18] due to the challenge of integrating multiple modalities. In this study, combining deep learning (DL) techniques and survival analysis, we built end-to-end dementia risk prediction models integrating 3D magnetic resonance imaging (MRI) images, together with genetic and demographic features.

To demonstrate the dementia risk prediction performance of our method, we conducted model comparison across different architectures, inputs, as well as different age groups and follow-up time horizons. We tested models on Rotterdam Study and external validated on Alzheimer’s Disease Neuroimaging Initiative (ADNI) data. In general, our deep survival neural networks with brain MRI images outperformed the baseline linear Cox Proportional Hazards (CPH) models with demographics and genetic data. This indicates our models’ ability to handle high-dimensional image data, and extract useful information beyond sex, age, genomics from images. In the same time, the neural network methods further demonstrated their capability of dealing with high-dimensional genetic data when raw single nucleotide polymorphisms (SNPs) were used as input (Model 5). We also demonstrated the utility of applying a pre-trained model, which originally trained for predicting brain age [3], to our tasks as a means to enhance prediction accuracy. Although our models did not surpass extended CPH models with MRI markers (total brain volume, hippocampal volume, white matter (WM) hyperintensity volume, and presence of lacunes/cortical infarcts), this result is not entirely unexpected given that these MRI markers were well-extracted pre-recognized risk factors. Nevertheless, we found our models outperformed extended CPH models in external validation among young subjects (60–69 age group) and were most suitable in short-term follow-up prediction (3-year) on Rotterdam Study from the stratified analysis. Furthermore, the combination of MRI image features and MRI markers yielded the best prediction performance in most scenarios, suggesting complementary information from MRI images for dementia risk prediction.

DL tools are often referred to as “black boxes” as they exhibit opacity in terms of how they arrive at their outputs. While most DL studies focus on predictive power, understanding the underlying explanations of models is frequently overlooked. And models that align with prior human knowledge are more likely to be adopted in medical practice, as such models tend to be safer and more reliable in unseen scenarios. Another primary innovation of our study is to provide comprehensive explanation of deep survival model predictions. We used Gradient-weighted Class Activation Mapping (Grad-CAM) [27] to post-hoc calculate the attention of our models on MRI images on voxel- and region-level respectively. Furthermore, we used DeepSHAP [28] to post-hoc calculate the feature importance for prediction. We showed that the brain regional and SNP importance calculated from one iteration (of the 5-fold cross-validation) were generally aligned with the average importance calculated from five iterations, suggesting the robustness of importance explanation. Among the top 10 important brain regions consistently highlighted by our models, the volumes of amygdala, hippocampus, thalamus and pallidum have been reported significantly associated with dementia risk, while the volumes of accumbens and putamen were found significant when interacting with APOE-ε4 in a previous study [5]. Similarly, among the top 5 important SNPs identified by DeepSHAP, rs10437655 (*SPI1*), rs62374257 (*COX7C*) and rs7912495 (*USP6NL*) were linked to Braak neurofibrillary tangles stage [29], and rs450674 (*MAF*) to neuritic plaques [30], both markers of Alzheimer’s disease neuropathologic changes (ADNC). Whereas rs6943429 (*UMAD1*) was found to be significantly associated with higher cognitive resilience [31]. Interestingly, the *UMAD1* gene was identified as a novel gene through the method of survival analysis in a recent research [32]. Furthermore, the SNP importance seems to have a positive relationship with the stage II replication meta-analysis significance (notably when p-value > 0.01) reported from the GWAS results of Alzheimer’s disease [33] (**Figure S9.2**), and seems to have a positive correlation with the variance of SNP alleles observed in the population (**Figure S9.3**). Among the SNPs mostly interacted with brain regions, their mapped genes: *ANKH* (rs112403360, No.11), *UMAD1* (rs6943429, No.21), *CTSH* (rs12592898, No. 53) and *CTSB* (rs1065712, No.28) are highly expressed in almost all brain structures according to the Allen human brain atlas [34] (**Figure S9.4**). rs112403360 was also identified associated with hippocampal sclerosis [30] and its protective allele was found most strongly enriched in cognitively healthy centenarians [35]. Besides, rs72824905 (No. 59) in *PLCG2* gene was found significantly associated with reduced volume of pallidum and putamen [36]. These consistencies with previous researches further validate our method. Finally, we plotted the feature interaction for prediction and estimated the interaction strengths, revealing notable interactions between imaging and genetic features in the risk prediction. This highlights the significance of integrating the two modalities for dementia risk prediction, and such explanation may provide insights for further exploration of disease etiology.

Several other contributions of this study warrant brief consideration. Firstly, current deep learning dementia prediction studies have mostly predicted conversion based on classification models [11, 37, 38]. These models ignore the information of time to conversion and will dramatically decrease the training sample size when in long-term prediction due to the requirement for sufficient follow-up time. Our models based on survival analysis makes full use of time-to-event information and right-censored samples. Secondly, while previous research mainly focused on short-term prediction [39], with conversion from mild cognitive impairment (MCI) to dementia [37], or from normal to MCI [38], we extend those findings to long-term follow-up from cognitively healthy to dementia. Last but not least, we discussed three often overlooked but critical issues in dementia prediction research: the influence of imbalanced data, age of included samples, and MCI patients, which adds valuable insights into study design for future studies.

The limitations of this study are as follows. First, we did not optimize our deep survival models on the feature numbers of imaging and genetic data. There is still room for improvement in the model architecture. Second, despite the substantial cohort size of the Rotterdam Study, DL models generally benefit from larger sample sizes, particularly for more dementia cases. Third, unlike traditional statistical models, our models still lack the inherent interpretability and do not provide significance indicators like p-values. Furthermore, stricter t-test could be performed considering the randomness of the training set in cross-validation [40]. Last but not least, our models did not incorporate WM, which is typically analyzed using T2-FLAIR MRI images. A recent study showed that WM signature clusters are differentially associated with dementia [41]. Genetic association findings were also empowered with the combination of T1 and T2-FLAIR imaging endophenotypes [42]. This suggests WM images may help with predicting dementia together with genetic data as well, while the challenge still lies in integration strategy and the computation complexity.

In conclusion, this study stems from the challenges and potential of leveraging multi-domain, high-dimensional data - including 3D MRI images, genetic data, and demographic characteristics - to enhance dementia risk prediction. Our innovative approach demonstrates the viability of using deep learning models to integrate these data, with reliable explanation. While this work represents an initial attempt, several areas require improvement and further research. Future efforts could benefit from exploring different deep learning model architectures, investigating practical strategies like expanding to multi-cohort datasets, federated learning and transfer learning to optimize model and enhance generalizability. With the advantage of not relying on pre-extracted features, our method will benefit a lot from larger sample size and more diverse samples. Beyond MRI and genomics, it holds potential for integration with other data types, advancing predictive accuracy and understanding of dementia etiology. By doing so, we can move closer to the goal of precision medicine, where individualized risk predictions, informed by comprehensive data analyses, become a reality.

## 4. METHODS

### 4.1 Study population

Data used for training and testing were obtained from the Rotterdam Study, a large prospective population-based cohort study that addresses determinants and occurrence of cardiovascular, neurological, ophthalmologic, psychiatric, and endocrine diseases in the elderly. At present the Rotterdam Study incorporates four cohorts that were established in 1989, 2000, 2006, and 2015 respectively. For each cohort, follow-up examinations take place every 3–4 years. By the end of 2020, 17931 subjects (residents aged 40 years or over, residing in Ommoord, a district of Rotterdam, the Netherlands) had participated in the study. From August 2005 onwards, a dedicated 1.5T MRI scanner has been operational in the research center [43].

**Figure 5:**
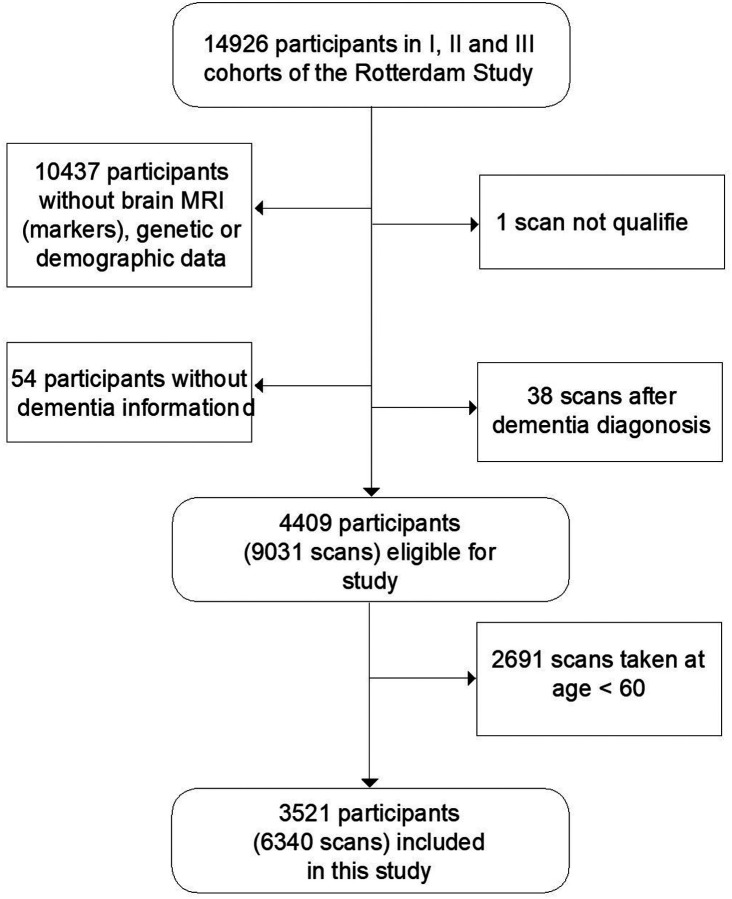
Data selection process of the Rotterdam Study data included in the study Acronyms: magnetic resonance imaging (MRI)

Analyses were based on the first three cohorts in the Rotterdam Study (14,926 subjects, aged 45 years or over). Longitudinal scans for each subject were included to capture changes over time, which also naturally increased our number of training images. We excluded individuals who did not have complete brain MRI, genetic or demographic data (date of birth and sex) (N = 10437). We also excluded individuals who lacked information on dementia or for whom the available information was unusable at baseline and during follow-up examinations (N = 54). Furthermore, we excluded MRI scans that did not pass quality check (S = 1) (See 4.4) and scans taken after dementia diagnosis (S = 38). This resulted in 4409 participants with 9031 scans eligible for study. Based on the age distribution plot in **S1**, a negligible number of incident dementia cases were present during the follow-up period on scans taken under the age of 60. Therefore, we excluded scans taken under the age of 60 (S = 2691) in the end. An experiment further supporting this exclusion in our study can be seen in **S5**. Finally, 3521 subjects with 6340 scans were included in this study. The selection process is shown in Fig. 5.

Data used for the external validation were obtained from the Alzheimer’s Disease Neuroimaging Initiative (ADNI) database. ADNI was launched in 2003 as a public–private partnership aimmed to determine whether serial magnetic resonance imaging (MRI), positron emission tomography (PET), other biomarkers, and clinical and neuropsychological assessments could be combined to track the progression of mild cognitive impairment (MCI) and early Alzheimer’s disease. Current objectives include validating biomarkers for clinical trials, enhancing cohort diversity to improve generalizability, and providing comprehensive data on Alzheimer’s disease diagnosis and progression to the research community. For detailed data information, see adni.loni.usc.edu, and is not further elaborated below. All risky samples with complete data described above were included.

### 4.2 Ethics statement

The Rotterdam Study has been approved by the Medical Ethics Committee of the Erasmus MC and by the Ministry of Health, Welfare and Sport of the Netherlands, implementing the Wet Bevolkingsonderzoek: ERGO (Population Studies Act: Rotterdam Study). All participants provided written informed consent to participate in the study and to obtain information from their treating physicians. All research procedures involving human participants were performed in accordance with the Declaration of Helsinki and relevant guidelines and regulations.

### 4.3 Imaging data

We used 3D T1-weighted images for subsequent analyses. A 1.5T MRI scanner (General Electrics Healthcare, Milwaukee, WI, USA) was used to acquire data from the participants of the Rotterdam Study. Details of the brain MRI scan protocol were described in [44, 45]. 1.5T or 3T scanners were used to acquire data from the participants of the ADNI, as described on ADNI website. We selected four candidate brain MRI markers (total brain volume, hippocampal volume, white matter (WM) hyperintensity volume, and presence of lacunes or cortical infarcts) according to a previous study, which reported a further improvement of dementia risk prediction [13]. Total brain volume was calculated on T1-weighted scans using an in-house developed KNN-segmentation method in a previous study, which was defined as the sum of total WM and total GM volume [46]. Hippocampal volume was derived from T1-weighted scans using FreeSurfer 6.0, which automatically segmented cortical and subcortical structures. WM hyperintensity volume was calculated on T2-weighted fluid-attenuated inversion recovery (FLAIR) scans in a previous study, by summing the volumes of all WM lesions detected [44]. For the presence of infarcts, lacunes and cortical infarcts were scored following the Rotterdam Scan Study protocol [47].

### 4.4 Image preprocessing

GM MRI images were segmented out as interests in this study. We performed voxel-based morphometry (VBM) according to an optimized protocol [48, 49]. First, GM were segmented from T1-weighted brain MRI scans based on the FreeSurfer segmentation results. The Brain-stem part was not included. Second, all GM maps were nonlinearly registered to the standard Montreal Neurological Institute GM probability template (ICBM 152 Nonlinear atlases version 2009) with 1 × 1 × 1 mm voxel resolution. Finally, a spatial modulation procedure was performed on the registered GM maps to avoid differences in absolute volume due to the registration. This was achieved by multiplying voxel density values by the Jacobian determinants estimated during registration. As smoothing is a subgroup of possible mathematical operations which the network filters in the convolutional layer can represent, we did not apply smoothing on the VBM results. The matrix size of the VBM results was 196 × 232 × 188. After VBM processing, we performed a quality control to exclude images with more than 5% outlier voxels (outlier was defined as falling within the top or bottom 5% of the distribution) and an additional visual check to exclude the outliers. 4 out of 12491 images did not pass the quality control. We applied cropping and padding on the images to cut proper empty edges, and masked the images with a GM mask. The matrix size of the final GM MRI images input to the models was 160 × 192 × 144.

### 4.5 Genetic data

The Rotterdam Study Genotyping platform consists of Illumina 550 duo and Human610-Quad BeadChip. Details of the genotyping, quality control and imputation were described previously [50]. ADNI Genotyping was performed using the Human610Quad BeadChip (Illumina, Inc., San Diego, CA). We selected genetic variations based on the most recently published genome-wide association study (GWAS) results of Alzheimer’s disease [33]. According to the GWAS results, we extracted allele numbers of *APOE*-ε4 genotype and 76 SNPs (**Table S3**) out of 83 risk SNPs [33] which are available in the Rotterdam Study genotype data. We also calculated PRS with the extracted SNPs. *APOE*-ε4 was not included in the PRS calculation.

### 4.6 Assessment of dementia

Participants were screened for dementia at baseline and during follow-up examinations using a three-step protocol. Initially, cognitive screening was performed with the Mini-Mental State Examination (MMSE) and the Geriatric Mental Schedule (GMS) organic level. Screen-positive individuals (MMSE < 26 or GMS organic level > 0) underwent further evaluation with the Cambridge Examination for Mental Disorders in the Elderly. Additionally, interval diagnoses in between visits were monitored through computerized linkage with digitized medical records. When available, clinical neuroimaging data were also incorporated into the diagnostic process; data from research scans was not used for diagnosis. Finally, dementia diagnosis was established according to the standard criteria for dementia (DSM-III-R) and Alzheimer’s disease (NINCDS-ADRDA) by a consensus panel led by a consultant neurologist [2, 51]. The incident dementia assessment used in this study was followed up until 1st Jan, 2020.

### 4.7 Deep survival neural networks

**Figure 6:**
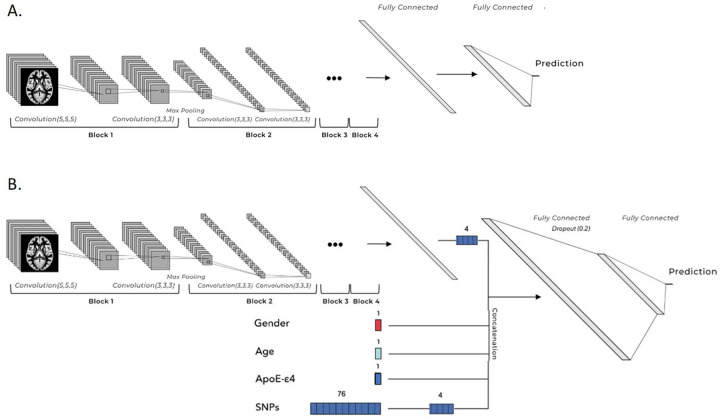
Architectures of deep survival neural networks used in the study. A. Deep survival neural network with only image inputs. B. Deep survival neural network with additional tabular inputs. Acronyms: single nucleotide polymorphism (SNP)

We developed our deep survival models combining the CNN and CPH model. The architecture of the network as illustrated in Fig. 6A was inspired by [3, 16]. For the CNN part, four convolutional blocks with 32, 48, 64, 80 kernels and a 2 × 2 × 2 max pooling layer respectively were expected to extract valuable features from MRI images. Then, two fully-connected layers with 32 and 4 nodes connected every feature to a single output neuron to generate risk predictions, which were designed to increase the network’s capacity of interweaving information. A dropout strategy with a rate of 0.2 was used between two fully-connected layers in order to avoid overfitting. The Rectified Linear Unit (RELU), a non-linear activation function, was set in all intermediate layers, from which non-linear interactions among features could be detected. The linear activation function was used in the output layer in order to give continuous predictions. The prediction outcome estimated the log-hazard ratio for each subject (see Eq. 1). Therefore, higher predictions indicate higher risk to develop dementia.

When combining tabular input features to the network, all features were concatenated before the first fully-connected layer. This enabled the network to truly view image information in the context of the tabular information. Especially, all continuous variables were normalized when input in the models to ensure proportionate weight updates, and we set the numbers of imaging and genetic feature nodes to the same (4 features) before concatenation, so as to avoid the extreme dimensionality imbalance and to capture sufficient information among different modalities. As mentioned in the literature, this imbalance will implicitly steer the network to focus on high dimensional features during training while underusing other features [52]. An example of the network architecture is illustrated in Fig. 6B.

For the loss function of the network, according to [16], a negative log transformation of the partial likelihood estimator of the linear CPH model was used (see Eq. 3–4). The equation of a CPH model with explanatory variables *X*_1_… *X*_k_ parameterized by β is defined as Eq. 1:

1 $$log\;(h_{i}(t)/h_{0}(t))=\beta_{1}x_{i1}+\beta_{2}x_{i2}+\ldots +\beta_{k}x_{ik}$$

2 $$h\left(t\right)\overset{\text{def}}{=}P(t\leq T<t+\Delta t|T\geq t)/\Delta t$$

Where *i* indicates individual *i*, *h*_*i*_ (*t*) is the hazard of event for individual *i* at time *t* based on *x*_*i*1_… *x*_*ik*_, which is a conditional density of the probability of event occurring at time *t*, given that the event has not yet occurred prior to time *t* (Eq. 2). *h*_0_ (*t*) is the baseline hazard at time *t* when all explanatory variables *x* are set to 0. The CPH model is under the assumption that the hazard of subjects and baseline are proportional across time.

The parameters *β* are estimated by maximizing the partial likelihood, which enlarges the risk difference between each individual who develops event and the individuals still at risk at the corresponding follow-up time:

(3) $$L_{c}(\beta )=\prod_{i:e_{i}=1}\frac{h_{0}\left(t_{i}\right)\text{exp}\left({\hat{\beta }}^{T}x_{i}\right)}{\sum_{j\in R\left(t_{i}\right)}h_{0}\left(t_{i}\right)\text{exp}\left({\hat{\beta }}^{T}x_{j}\right)}=\prod_{i:e_{i}=1}\frac{\text{exp}\left({\hat{\beta }}^{T}x_{i}\right)}{\sum_{j\in R\left(t_{i}\right)}\text{exp}\left({\hat{\beta }}^{T}x_{j}\right)}$$

Where *e*_*i*_, *t*_*i*_, *x*_*i*_ are the respective event indicator, time-to-event, and values of variables for individual *i*. The event indicator represents whether an individual developed dementia or not. The time-to-event is the shorter duration obtained by subtracting the date of scan from both the date of diagnosis and the end date of the study, while the latter one indicates a censored sample. The risk set *R* is the set of individuals still at risk at time *t*_*i*_.

Therefore, with our model prediction output ${\overset{ˆ}{y}}_{\theta }$ (*x*), our loss function was of equation:

4 $$L_{ds}(\theta )=-\text{log}\left(L_{c}(\theta )\right)=-\sum_{i:E_{i}=1}\left({\overset{ˆ}{y}}_{\theta }\left(x_{i}\right)-\text{log}\sum_{j\in R\left(T_{i}\right)}\text{exp}\left({\overset{ˆ}{y}}_{\theta }\left(x_{j}\right)\right)\right)$$

Our model parameters *θ* were optimized by minimizing *L_ds_*.

For the evaluation metric of the network, we measured the concordance-index (C-index) [53]. The C-index is the probability of the concordant predictions in pairs in the set of all comparable pairs, admissible if the shorter time-to-event is an event. A C = 0.5 is equivalent to a random prediction whereas C = 1.0 is a perfect ranking of time-to-event. Our best models were saved based on maximum validation C-index during model training.

(5) $$C=\frac{\sum_{i\neq j}I\left({\overset{ˆ}{y}}_{i}>{\overset{ˆ}{y}}_{j}\right)I\left(t_{i}<t_{j}\right)I\left(e_{i}=1\right)}{\sum_{i\neq j}I\left(t_{i}<t_{j}\right)I\left(e_{i}=1\right)}$$

### 4.8 Model comparison and stratified analysis

In order to evaluate the performance of our models with MRI images, we used baseline linear CPH models with no brain MRI input, extended linear CPH models with brain MRI markers (mentioned in 4.3), as well as DeepSurv models from [16] representing the nonlinear version of the extended linear CPH models as comparison. For our deep survival models, two training strategies were compared, one was fine-tuning the CNN part based on the pre-trained model on age prediction [3], another was training from scratch. We also assessed whether incorporating MRI images added value to dementia risk prediction in addition to MRI markers, by combining MRI markers with image features in linear models. The image features were derived from deep survival (fine-tuned) model with MRI images only.

Furthermore, we explored how gradually input sex, age, genetic data influenced the model performance. Therefore, five models with different inputs in addition to MRI inputs were compared:

Model 1: Only brain MRI inputs

Model 2: Age + Sex + MRI inputs

Model 3: Age + Sex + *APOE*-ε4 + MRI inputs

Model 4: Age + Sex + *APOE*-ε4 + PRS + MRI inputs

Model 5: Age + Sex + *APOE*-ε4 + SNPs + MRI inputs

We also performed stratified analysis on age groups of 60–69, 70–79 and 80–89 years as well as on follow-up time of 3-, 5-, and 10-years. We therefore further evaluated models’ discriminative abilities on different scenarios of age and follow-up time.

To take account of model robustness in the comparison, we performed a stratified 5-fold cross-validation. The mean C-index and 95% confidence intervals across the five iterations were reported. Pair-wised t-test was performed to assess the significance of performance differences.

### 4.9 Data splitting

We split the data by stratified sampling based on dementia diagnosis and *APOE*-ε4. For each iteration, we used one of the five folds as the test set (20%) and remaining for training (80%). The training data was then stratified, with 10% sampled as validation set. Images from the same subjects were included only in one set to avoid data leakage. Participants progressing from MCI to dementia were not included in the test set as we wanted to evaluate the predicting of progression from normal subjects. We only included one image per subject in the test set for evaluation. Repeated scans from the same subject taken in a short period were used for a reproducibility test.

### 4.10 Extra experiments

To optimize our study samples, we performed three extra experiments. First, as mentioned in 4.1, we experimented with whether or not to include samples under the age of 60. Although it is fairly common in dementia cohort studies to exclude these samples, in DL settings which benefit from larger samples size, we wanted to test whether their brain images still be useful for improve model performance.

Second, regarding the imbalanced data problem, which may result in inaccurate risk predictions among individuals who are more likely to develop an event when case proportion is low [54, 55], we did experiments on using different case-control proportions in training.

Last, we experimented with the inclusion strategies for MCI samples (i.e. individuals who have ever been diagnosed with MCI). Since MCI is an intermediate state between normal and dementia, their scans may appear closer to the dementia class [52], making binary dementia labels (0/1) inaccurate for reflecting their conditions and potentially affecting model training. However, their image data can still provide useful information on progression clues. Therefore, we compared three different inclusion strategies on MCI samples in the model training: excluding all MCI samples, only excluding control samples with MCI but including MCI samples progressed to dementia, and including all MCI samples.

More details and experiment results are described in **S5-S7**. Based on the experiment results, we excluded samples under the age of 60 in this study, maintained the real case-control distribution, and excluded control samples with MCI from the training set (but still included them in the test set).

### 4.11 Model explanation

Model explanations for Model 5 were performed to evaluate whether Image- and SNP-derived features identified by the deep survival model correspond to previous dementia-associated findings, and whether the features contribute to the prediction independently or through interactions across data modalities.

Firstly, we evaluated the feature importance for the risk prediction. Gradient-weighted Class Activation Mapping (Grad-CAM) [27] was used to visualize our model attention by heat map on 3D images based on Rotterdam Study test set. Grad-CAM calculates the gradients of outcome flowing into the convolutional layer in CNNs and uses an activation function to locate the specific area of model focus. We used the second convolutional layer in CNN for activation mapping thereby generating a smaller localization scope. Based on the Grad-CAM results, we inspected the brain region importance by calculating the mean voxel attention intensity within each anatomically defined region based on the Hammersmith atlas [56]. Furthermore, DeepSHAP was used to compute the feature importance from the concatenate layer as well as the importance of SNPs, chosen for the combined benefits of DeepLIFT and Shapley values [28]. DeepSHAP estimates additive feature attributions (SHAP values) to the outcome for each individual sample, where the mean absolute SHAP values of all individuals indicates the feature importance. We used the Rotterdam Study training set as the reference dataset providing background distribution for computation and applied the DeepSHAP explainer on the Rotterdam Study test set.

Secondly, we explored the feature interaction patterns in the risk prediction. We used the statistical interaction detection method from [29] to quantify the interaction strength. Combining the neural network gradient of the imaging feature and genetic feature to each brain region and SNP, respectively, we further mapped the interaction strength to all pairs of SNPs and brain regions. Interactions could also be intuitive to see where the effect of one feature on the outcome depends on the value of another feature. Therefore, we also plotted the interaction relationships between pairs of the features based on the SHAP values.

## Supplementary Material

This is a list of supplementary files associated with this preprint. Click to download.

- SupplementarymaterialsSR.docx

## Figures and Tables

**Table 1:** Population characteristics summary of the Rotterdam Study data included in the study and in one fold of the 5-fold cross-validation

	Total	Training^#^	Validation^#^	Test	External Validation
N_subj_*	3521	2371	263	726	515
N_subj_ dementia	258	194	22	42	170
N_img_^†^	6340	4227	513	726‡	515
N_img_ dementia	386	287	30	42‡	170
TTE (years), mean ± sd	7.75 ± 3.15	7.73 ± 3.14	7.94 ± 3.06	7.16 ± 3.07	8.17 ± 4.52
Sex (F/M), prop.	0.54 / 0.46	0.54 / 0.46	0.56 / 0.44	0.55 / 0.45	0.61 / 0.39
Age (years), mean ± sd	68.74 ± 7.76	68.39 ± 7.61	69.08 ± 8.22	70.36 ± 8.51	75.36 ± 6.47
*APOE*- ε4 (0/1/2), prop.	0.72/0.26/0.02	0.72/0.26/0.02	0.74/0.22/0.04	0.72/0.25/0.02	0.54/0.37/0.09
PRS^§^, mean ± sd	3.33 ± 0.21	3.32 ± 0.21	3.34 ± 0.21	3.34 ± 0.22	3.40 ± 0.22
TBV/ICV, mean ± sd	0.81 ±0.04	0.82 ± 0.04	0.82 ± 0.04	0.81 ±0.04	0.64 ± 0.04
HV/ICV^§^, mean ± sd	0.59 ± 0.05	0.59 ± 0.05	0.59 ± 0.06	0.58 ± 0.05	0.25 ± 0.04
WMHV/ICV^§^, median [min, max]^¶^	0.34 [0.02,8.98]	0.33 [0.02,8.98]	0.38 [0.04,7.97]	0.38 [0.02,7.64]	0.28 [0.10,2.14]
Infarcts (0/1), prop.	0.89/0.11	0.90/0.10	0.89/0.11	0.89/0.11	0.92/0.08

* Number of subjects

† Number of images

‡ Selection only includes one image per subject

§ Multiplied by 100

¶ Median and range are presented because of skewed distributions

# Excluding control samples with MCI

Acronyms: time-to-event (TTE), polygenic risk score (PRS), intracranial volume (ICV), total brain volume (TBV), hippocampal volume (HV), white matter hyperintensity volume (WMHV)

**Table 2:** Dementia risk prediction performance of linear CPH models, DeepSurv and deep survival models with different model inputs. Mean C-index and 95% confidence intervals are based on 5-fold cross-validation. (A) Test results on Rotterdam Study. (B) External validation results on ADNI.

(A)							
Model C-index (95% Cl)	Linear CPH model			DeepSurv	Deep Survival model		
	Baseline	Extended			TFS		Fine-tune
	None	TBV, HV	MRI markers*	MRI markers	MRI images	MRI images	Image features + MRI markers
Model 1	-	0.85 (0.84, 0.86)	0.86 (0.85, 0.88)	0.86 (0.84, 0.87)	0.84 (0.80, 0.88)	0.86 (0.82, 0.89)	0.88 (0.85, 0.90)
Model 2	0.83 (0.81, 0.85)	0.87 (0.86, 0.88)	0.87 (0.86, 0.89)	0.87 (0.86, 0.88)	0.85 (0.83, 0.88)	0.85 (0.82, 0.88)	0.88 (0.86, 0.90)
Model 3	0.85 (0.84, 0.85)	0.88 (0.87, 0.88)	0.88 (0.88, 0.89)	0.87 (0.85, 0.88)	0.86 (0.85, 0.88)	0.87 (0.85, 0.89)	0.89 (0.87, 0.91)
Model 4	0.85 (0.85, 0.86)	0.89 (0.88, 0.90)	0.89 (0.88, 0.90)	0.88 (0.86, 0.89)	0.86 (0.84, 0.87)	0.88 (0.85, 0.90)	0.90 (0.88, 0.92)
Model 5	0.81 (0.77, 0.84)	0.85 (0.81, 0.88)	0.85 (0.80, 0.90)	0.86 (0.84, 0.87)	0.86 (0.82, 0.90)	0.86 (0.84, 0.88)	0.87 (0.82, 0.91)

(B)					
Model C-index (95% CI)	Linear CPH model			Deep Survival model	
	Baseline	Extended		Fine-tune	
	None	TBV, HV	MRI markers*	MRI image	Image features + MRI markers
Model 1	-	0.66 (0.66, 0.66)	0.65 (0.65, 0.65)	0.65 (0.65, 0.66)	0.66 (0.65, 0.67)
Model 2	0.47 (0.47, 0.47)	0.59 (0.58, 0.60)	0.60 (0.59, 0.61)	0.58 (0.55, 0.61)	0.65 (0.62, 0.67)
Model 3	0.56 (0.56, 0.57)	0.65 (0.64, 0.65)	0.66 (0.65, 0.67)	0.63 (0.63, 0.64)	0.68 (0.67, 0.70)
Model 4	0.58 (0.58, 0.59)	0.65 (0.64, 0.66)	0.66 (0.65, 0.67)	0.63 (0.61, 0.64)	0.69 (0.68, 0.70)
Model 5	0.57 (0.55, 0.59)	0.62 (0.61, 0.64)	0.63 (0.61, 0.65)	0.61 (0.59, 0.64)	0.66 (0.64, 0.68)

Model 1: Only brain MRI inputs (as indicated by table columns)

Model 2: Age + Sex + MRI inputs (as indicated by table columns)

Model 3: Age + Sex + *APOE*-ε4 + MRI inputs (as indicated by table columns)

Model 4: Age + Sex + *APOE*-ε4 + PRS + MRI inputs (as indicated by table columns)

Model 5: Age + Sex + *APOE*-ε4 + SNPs + MRI inputs (as indicated by table columns)

* MRI markers indicate the TBV, HV, WMHV and lacunes/cortical infarcts.

Acronyms: confidence interval (CI), Cox Proportional Hazards (CPH), fully-connected neural network (FCNN), total brain volume (TBV), hippocampal volume (HV), white matter hyperintensity volume (WMHV), magnetic resonance imaging (MRI), gray matter (GM); polygenic risk score (PRS), single nucleotide polymorphism (SNP)

## Data Availability

Data used in external validation of this article were obtained from the Alzheimer’s Disease Neuroimaging Initiative (ADNI) database (adni.loni.usc.edu). As such, the investigators within the ADNI contributed to the design and implementation of ADNI and/or provided data but did not participate in the analysis or writing of this report. A complete listing of ADNI investigators can be found at: [https://adni.loni.usc.edu/wp-content/uploads/how_to_apply/ADNI_Acknowledgement_List.pdf] All ADNI data are shared through the LONI Image and Data Archive (IDA), a secure research data repository. Interested scientists may obtain access to ADNI imaging, clinical, genomic, and biomarker data for the purposes of scientific investigation, teaching, or planning clinical research studies. Access information can be found on [https://adni.loni.usc.edu/data-samples/adni-data/#AccessData].
